# A machine learning approach to genome-wide association mapping of disease resistance and geographic origin in sorghum

**DOI:** 10.1186/s12870-026-08468-z

**Published:** 2026-02-28

**Authors:** Ezekiel Ahn, Insuck Baek, Sunchung Park, Louis K. Prom, Seunghyun Lim, Jae Hee Jang, Seok Min Hong, Moon S. Kim, Lyndel W. Meinhardt, Clint Magill

**Affiliations:** 1 United States Department of Agriculture, Sustainable Perennial Crops Laboratory, Agricultural Research Service, United States, Beltsville, MD 20705 USA; 2 United States Department of Agriculture, Environmental Microbial and Food Safety Laboratory, Agricultural Research Service, United States, Beltsville, MD 20705 USA; 3 United States Department of Agriculture, Insect Control and Cotton Disease Research, Agricultural Research Service, Southern Plains Agricultural Research Center, United States, College Station, TX 77845 USA; 4 Department of Civil Urban Earth and Environmental Engineering, Ulsan National Institute of Science and Technology, UNIST-gil 50, Ulsan, 44919 Republic of Korea; 5 Department of Plant Pathology and Microbiology, Texas A&M University, College Station, TX 77843 USA

**Keywords:** Genetic diversity, GWAS, Anthracnose, Head smut, Downy mildew, Mini-core collections, Landraces

## Abstract

Sorghum, the fifth most important cereal crop globally, faces persistent threats from fungal diseases that limit productivity and resilience. To investigate the genetic basis of disease resistance and geographic adaptation, we applied a machine learning-enabled genome-wide association study (GWAS) to a panel of 377 genetically diverse sorghum accessions, incorporating nearly 300,000 SNP markers and phenotypic evaluations for resistance to anthracnose, head smut, and downy mildew. While disease resistance phenotypes did not cluster strictly by geographic origin, SNP-based analyses revealed significant genetic differentiation among accessions from different regions, particularly involving a genetically distinct group from Senegal. Bootstrap Forest models highlighted candidate SNPs predictive of geographic origin, most notably on Chromosome 10, near genes encoding transcription factors (e.g., bHLH, EREBP-like) and DUF6598-domain proteins with potential roles in plant defense. For disease resistance, top-ranked SNPs were located near genes implicated in canonical immune pathways, including zinc-binding proteins (anthracnose), NB-ARC and LRR-containing proteins (head smut), and F-box proteins (downy mildew). Although exploratory in nature, these findings suggest that local adaptation to pathogen pressure may have shaped sorghum’s genomic landscape. The identified candidate genes and associated SNPs help prioritize targets for marker-assisted selection and follow-up functional validation, contributing to the development of sorghum varieties with enhanced resistance and adaptability.

**Supplementary Information:**

The online version contains supplementary material available at 10.1186/s12870-026-08468-z.

## Introduction

Sorghum (*Sorghum bicolor* L. Moench) is a staple cereal crop for over 500 million people worldwide, particularly in the semi-arid tropics of Africa and Asia, where it serves as a critical source of food, feed, and biofuel [1–3]. Sorghum’s high tolerance to drought and heat makes it an excellent candidate crop for ensuring food security in the future, particularly in regions susceptible to climate change [4]. However, its production is significantly constrained by both biotic and abiotic stresses [5]. Significant yield losses in sorghum can result from fungal diseases such as anthracnose, head smut, and downy mildew, posing a severe threat to farmers’ livelihoods and the food security of dependent populations [6]. Disease-resistant cultivars are often considered the best method for managing sorghum diseases [7]. Therefore, developing sorghum cultivars with durable disease resistance and broad adaptation remains a priority.

Anthracnose, caused by the fungal pathogen *Colletotrichum sublineola*, is a widespread and destructive sorghum disease, affecting all aerial parts of the plant and causing significant reductions in grain yield and quality [8]. Yield losses exceeding 50% have been reported under severe anthracnose epidemics [9, 10]. Head smut, caused by the biotrophic fungus *Sporisorium reilianum* (Kühn) Langdon & Fullerton, is another major constraint to sorghum production, particularly in humid and warmer growing regions [11]. Infection by *S. reilianum* leads to the replacement of the sorghum panicle with a large, black sorus filled with fungal spores, resulting in a complete loss of grain yield in infected plants [12]. Downy mildew, caused by the oomycete *Peronosclerospora sorghi*, can also result in substantial losses, particularly in susceptible varieties grown under humid conditions [13]. *P. sorghi* infection manifests as localized leaf lesions and systemic infection, leading to stunting, reduced tillering, and the development of characteristic “downy” abaxial growth on infected leaves [14]. Moreover, systemically infected seedlings turn pale yellow or exhibit light-colored streaking on their leaves, are chlorotic and stunted, and prematurely die [7]. While some sources of resistance to these diseases have been identified and deployed in sorghum breeding programs, new pathogen races and the complex, polygenic nature of resistance pose ongoing challenges [15, 16]. Further investigation is needed into the genetic architecture of resistance to these three diseases to develop sorghum varieties with durable and broad-spectrum resistance.

Sorghum is an ancient African crop that has spread across diverse environments, particularly in the semiarid tropics of Africa and South Asia [17]. The geographic origin of plants plays a pivotal role in shaping this diversity [18], as geographically distant populations have adapted to different selective pressures, including climate, soil types, and microbial populations. As an outcome, locally adapted landraces with unique trait combinations have evolved. Studies in sorghum and other crops have demonstrated that geographic patterns of genetic variation often mirror patterns of environmental variation, suggesting a strong link between adaptation and geographic origin [19–21]. For instance, accessions in arid regions may possess enhanced drought tolerance, while accessions from regions with high disease pressure have evolved unique and multiple resistance genes [22, 23]. Furthermore, humans’ historical movement and exchange of plant germplasm have further contributed to the complex patterns of genetic diversity observed in sorghum [24–26]. A deep understanding of how geographic origin has shaped sorghum’s genetic diversity is crucial for unraveling its evolutionary history and enhancing crop improvement. By analyzing the genomes of sorghum accessions from different regions, we can identify genes and pathways that have been selected under diverse environmental pressures. This knowledge can be directly applied to breeding more resilient and robust sorghum varieties.

The availability of diverse germplasm is also fundamental to understanding the genetic basis of adaptive traits and making significant progress in crop improvement [27–29]. Broadening the genetic base of breeding programs by including landraces and geographically diverse accessions can introduce novel alleles and allelic combinations that are not currently integrated into elite cultivars. This is particularly important for traits such as disease resistance, where pathogens continuously evolve and overcome existing resistance mechanisms [15, 16].

In 2001, the International Crops Research Institute for the Semi-Arid Tropics (ICRISAT) gene bank developed a core collection of 2,247 sorghum accessions from its larger germplasm collection, which comprised over 37,000 accessions [6, 30]. However, this core collection was too large for many replicated evaluation studies. Hence, a sorghum mini core collection consisting of 10% was developed to represent a global snapshot of sorghum diversity [6, 30]. This mini core collection has been widely used to study the genetic architecture of various traits such as disease resistance [31].

Based on this mini core collection, our previous study used a GWAS of resistance to anthracnose, head smut, and downy mildew [6]. The current study builds upon this foundation with two primary objectives. First, we characterized the genetic relationships among an expanded panel of 377 sorghum accessions, including the mini core collection and accessions from Senegal, to understand the population structure and identify potential sources of unique genetic variation. Second, we sought to identify genomic regions associated with both geographic origin and disease resistance using the machine learning model Bootstrap Forest (a variant of Random Forest) [32] for GWAS. The machine learning algorithm allows us to capture complex relationships between genetic markers and traits, providing a more nuanced understanding of the genetic architecture underlying these complex traits [33]. We hypothesized that this integrated approach, combining diverse germplasm, high-density SNP genotyping, and machine learning, would enable us to uncover novel genetic diversity, identify genomic regions underlying adaptation to different geographic origins, and pinpoint candidate genes associated with disease resistance. By leveraging the power of machine learning in GWAS, the results of this study provide a resource to prioritize loci for breeding and to guide follow-up functional studies toward improving sorghum resilience and productivity.

## Materials and methods

### Phenotypic and genotypic data

This study used phenotypic and genotypic data from a previous study [6]. A complete list of the 377 accessions used, along with their origins and phenotypic data, is provided in Supplementary Table 1. The 135 Senegalese accessions were obtained from the USDA-ARS Plant Genetic Resources Conservation Unit (Griffin, GA, USA) and were maintained and used in full compliance with relevant institutional, national, and international guidelines. These Senegalese accessions were not phenotyped for disease resistance and were included in this study solely for analyses of genetic diversity and geographic origin. For the mini core accessions, resistance to three major sorghum diseases, anthracnose, head smut, and downy mildew, was assessed under controlled greenhouse conditions. However, not all 242 accessions were evaluated for all three diseases. A total of 216 accessions were evaluated for anthracnose resistance using a spray inoculation method in a randomized block design with four replications. Accessions were classified as resistant or susceptible based on the presence of acervuli (fungal fruiting bodies). Downy mildew resistance was evaluated for 213 accessions using the sandwich inoculation technique in a randomized block design with three replications. This technique involved placing sprouted seeds between two infected leaf pieces on a wet filter paper in a Petri plate, with the pathogen’s spore-producing surface facing the seeds to facilitate infection [11]. Accessions with a disease incidence of 10% or less were considered resistant. Head smut resistance was assessed for 204 accessions using a syringe inoculation method in a randomized block design with three replications. Accessions were scored as resistant if no infection was detected. The 297,876 SNP markers used in this study were extracted from an integrated sorghum SNP dataset based on the sorghum reference genome version 3.1.1. The markers were originally generated using genotyping-by-sequencing (GBS) [6, 34–37]. Prior to analysis, the raw genotypic data was filtered, removing markers with a minor allele frequency (MAF) below 0.01 and a call rate of less than 90%. The remaining missing data were subsequently imputed using Beagle 4.1 [38].

### Multivariate analysis of phenotypic and genotypic data

Principal Component Analysis (PCA) and *t*-distributed Stochastic Neighbor Embedding (*t*-SNE) were performed using JMP Pro 17 software (SAS Institute Inc., Cary, NC) [39] to assess overall phenotypic variation and relationships among sorghum accessions. For the PCA, the binary classification for each disease (resistant/susceptible) was first converted into a one-hot encoded format to determine the contribution of each phenotype to the overall variation. *t*-SNE, a non-linear dimensionality reduction technique, was also employed with its default parameters (output dimensions = 2, perplexity = 30, maximum iterations = 1,000, initial principal component dimensions = 50, convergence criterion = 1e − 8, initial scale = 0.0001, Eta (learning rate) = 200, inflate iterations = 250, and random seed = 123) to further explore the underlying structure of the phenotypic variation and identify potential clusters of accessions with similar disease resistance profiles. Genetic distances among accessions were calculated based on the 297,876 SNP markers using the Identity by State (IBS) method implemented in TASSEL 5 software [40]. Two-tailed *t*-tests were performed to assess differences in mean pairwise IBS genetic distance between resistant and susceptible accessions for each disease. A one-way analysis of variance (ANOVA) was used to evaluate differences in genetic distances among groups based on their country and geographic region of origin.

We also performed a hierarchical clustering analysis using the Ward method based on SNP markers to investigate the genetic relationships among sorghum accessions, utilizing the default settings in JMP Pro 17.

### Genome-wide association analysis of geographic origin and disease resistance using Bootstrap Forest

We performed a GWAS using a machine learning approach based on Bootstrap Forest model to identify genomic regions associated with geographic origin and disease resistance. These models utilized the same 297,876 SNP markers and the phenotypic data, specifically the binary resistant/susceptible classification, for each geographic location and disease as the target variable. Unlike traditional GWAS methods that rely on linear or logistic regression and generate *p*-values based on statistical significance under a specific model, the Bootstrap Forest model does not directly produce *p*-values. Instead, model performance is assessed through measures like accuracy, and the contribution of individual SNPs is quantified using importance scores.

The Bootstrap Forest model, a variant of the Random Forest algorithm [32], was chosen for its ability to handle high-dimensional genomic data and capture complex, non-linear relationships between SNPs and traits [33]. A key advantage of this non-parametric approach is that it does not require the data to meet specific mathematical assumptions, such as normality, making it well-suited for our binary phenotypic classifications (resistant/susceptible) [33]. The analysis was performed using JMP Pro 17 using default settings unless otherwise specified [39]. For each model, 100 trees were grown with a bootstrap sample rate of 1 (meaning each tree was trained on a sample the same size as the original dataset, drawn with replacement). The importance score for each SNP, reported as ‘portion,’ is a measure of its contribution to the model’s predictive accuracy. It is calculated by summing the reduction in model error each time a particular SNP is used to split the data, and then averaging this across all trees in the forest [32, 33]. A higher score indicates that the SNP was more influential in correctly classifying accessions. It is important to note that unlike the linear models often used in traditional GWAS, this non-parametric approach does not calculate Phenotypic Variance Explained (PVE). The importance score is the analogous metric provided by the Bootstrap Forest model to quantify an SNP’s contribution to the trait.

#### Geographic origin

Bootstrap Forest models were trained to predict the geographic origin of the sorghum accessions, using either the country of origin or the broader geographic region as the target variable. The models were trained on the SNP markers, with the dataset randomly split (Random seed = 1 in JMP Pro 17) into 80% for training and 20% for validation. The data split was conducted using the default settings in JMP Pro 17, with the following parameters for the Bootstrap Forest models: number of trees in the forest = 100, number of terms sampled per split = 1, bootstrap sample rate = 1, minimum splits per tree = 10, maximum splits per tree = 2000, minimum size split = 5. We first assessed prediction accuracy on the training and validation sets to evaluate model performance. After that, we trained another model using the same settings but with 100% of the data for training, which allowed us to use the full dataset for identifying important SNPs while ensuring that the model had been adequately validated. The top SNPs associated with the country and region of origin were identified based on their importance scores (portion). To explore potential candidate genes involved in geographic adaptation, the single, physically closest gene to each top SNP was identified using the *Sorghum bicolor* reference genome v3.1.1 from Phytozome 12 (https://phytozome-next.jgi.doe.g.ov/*)* [41]. We did not apply a fixed search window; the exact distance to the nearest gene is reported in the results tables.

#### Disease resistance

The genetic basis of disease resistance was investigated using the same Bootstrap Forest modeling approach described for geographic origin. Separate models were trained to predict resistance to each disease: anthracnose, head smut, and downy mildew. These models used the same SNP markers and the phenotypic data (resistant/susceptible) for each disease as the target variable. Model parameters, data splitting procedures, and variable importance assessments were identical to those used in the geographic origin analyses. The top SNPs associated with resistance to each disease were identified based on their importance scores. Unlike traditional GWAS, which uses a statistical significance threshold (e.g., a *p*-value), a hard cutoff for the importance score was not applied in this exploratory analysis. Instead, top-ranking SNPs were selected based on a qualitative assessment of the Manhattan-like plots of SNP importance; we identified the most prominent signal peaks for each analysis and presented the lead SNPs, those with the highest importance scores within these peaks, as the most promising candidates for further investigation.

The nearest genes to these top SNPs were then identified using the *Sorghum bicolor* reference genome v3.1.1 from Phytozome 12 to explore potential genetic mechanisms underlying disease resistance [41]. A random seed of 1 was used for all machine learning model training to ensure reproducibility.

## Results

### Phenotypic and genetic diversity of disease resistance in the sorghum mini core collection

**Fig. 1:**
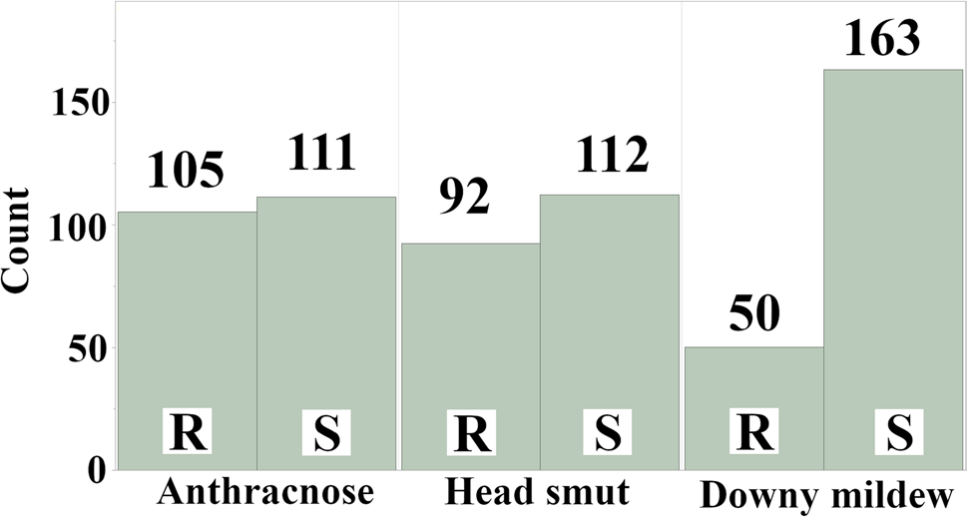
Counts of resistance and susceptibility to three diseases in a subset of the sorghum mini core collection. Resistance (R) and susceptibility (S) phenotypes are shown for anthracnose, head smut, and downy mildew. The 135 Senegalese lines were not phenotyped and were excluded from the graph. Not all accessions were evaluated for all three diseases. Sample sizes for each disease are as follows: anthracnose (*n* = 216), head smut (*n* = 204), and downy mildew (*n* = 213). Numbers above the bars indicate the number of accessions in each category (R or S)

We examined the counts of resistance and susceptibility phenotypes for each of the three diseases within the subset of the mini core collection that was phenotyped (Fig. 1). For anthracnose, 216 accessions were evaluated, with 105 classified as resistant and 111 as susceptible. Head smut resistance was assessed in 204 accessions, revealing 92 resistant and 112 susceptible lines. Downy mildew exhibited a distinct pattern, with 213 accessions evaluated, and a higher proportion of susceptible (163) than resistant (50) accessions.

**Fig. 2:**
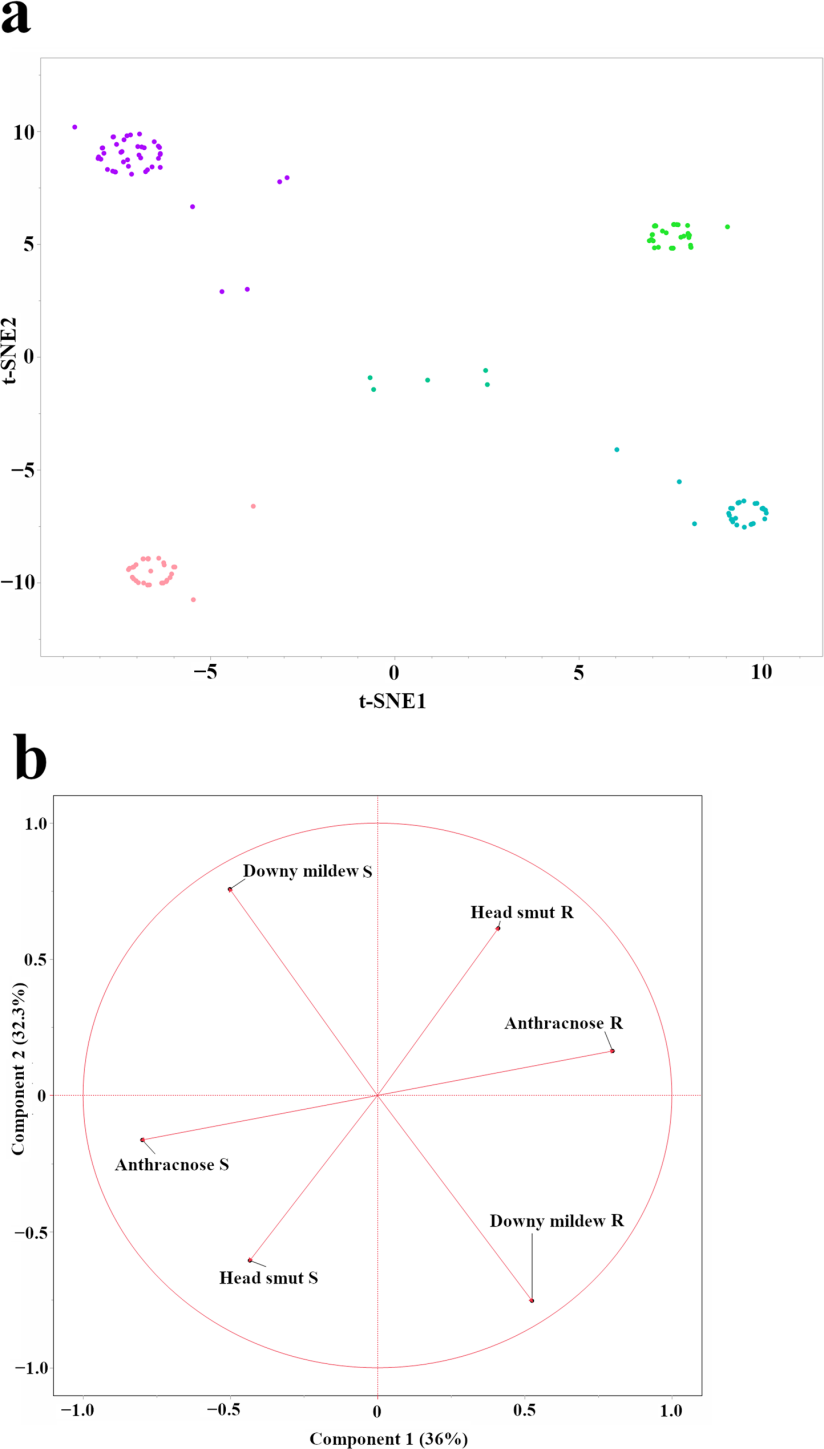
Global patterns of sorghum disease resistance and genetic diversity in the mini core collection. **a** *t*-SNE visualization of the phenotypic data (disease responses: resistant/susceptible), revealing four distinct clusters and potentially a fifth, more diffuse central group. **b** Loading plot showing the contribution of each disease resistance phenotype (resistance and susceptibility for anthracnose, head smut, and downy mildew) to the first two principal components (PC1 and PC2). Vector length and direction indicate the magnitude and direction of each variable’s influence. PC1 and PC2 explain 36% and 32.3% of the total phenotypic variance

To explore the phenotypic variation in disease resistance, a *t*-SNE analysis was performed (Fig. 2a). *t*-SNE visualization revealed four distinct clusters and a fifth, more diffuse central group. This pattern suggests four major phenotypic groupings and a fifth, more diffuse group with intermediate or mixed phenotypes, represented by the diffuse central cluster. It is essential to note that while the *t*-SNE analysis effectively groups the accessions into these local clusters, the global circular arrangement is an artifact of the visualization algorithm and lacks a specific biological interpretation. While these clusters show some degree of structure, they do not correspond directly to geographic origin, indicating that phenotypic similarity in disease resistance is not solely driven by geographic proximity.

The contributions of each disease resistance phenotype to overall phenotypic variation were further examined using a loading plot derived from a PCA (Fig. 2b). The first two principal components, PC1 and PC2, explained 36% and 32.3% of the total phenotypic variation, respectively. The loading plot showed that, for each disease, the resistance and susceptibility variables loaded in opposite directions (as expected for binary complements). While all three diseases contributed significantly to the observed variation, downy mildew resistance had the most substantial influence on PC2, whereas anthracnose resistance had the most substantial influence on PC1. This suggests that these two diseases have been major drivers of phenotypic divergence in the mini core collection.

Genetic diversity among the mini core accessions was assessed using SNP data. Genetic distances were calculated based on the IBS method (Fig. S1). When grouped by disease resistance phenotype, susceptible accessions exhibited slightly greater genetic distances compared to resistant accessions for anthracnose and head smut (two-tailed *t*-test: *p* = 0.0011 for anthracnose, *p* = 0.0196 for head smut). However, no significant difference was observed for downy mildew (*p* = 0.541). These results indicate that genetic distances differed modestly by phenotype for anthracnose and head smut, but not for downy mildew. We therefore refrain from inferring specific genetic architectures from IBS distances alone.

Analysis of genetic distances by country of origin revealed significant variation (one-way ANOVA: *p* < 0.0001). Notably, accessions from Nicaragua (0.13), Lesotho (0.138), and Thailand (0.14) exhibited the lowest genetic distances, while those from China (0.197), Algeria (0.198), Mali (0.206), Gambia (0.275), and Sierra Leone (0.284) exhibited the highest. These significant genetic distances are related to geographic region (ANOVA *p* < 0.0001), with accessions from South America (0.15), Southern Africa (0.151), and Central America & Caribbean (0.153) showing the lowest genetic distances, and those from the Middle East (0.191), West Africa (0.193), and East Asia (0.196) displaying the highest. Overall, mean IBS distances varied significantly across countries and regions, consistent with geographic structure in the genotyped panel. Similar resistance phenotypes observed across distant regions may reflect multiple historical processes, but we do not resolve these with the current data.

### Genetic relationships among mini core and Senegalese accessions based on SNP markers

**Fig. 3:**
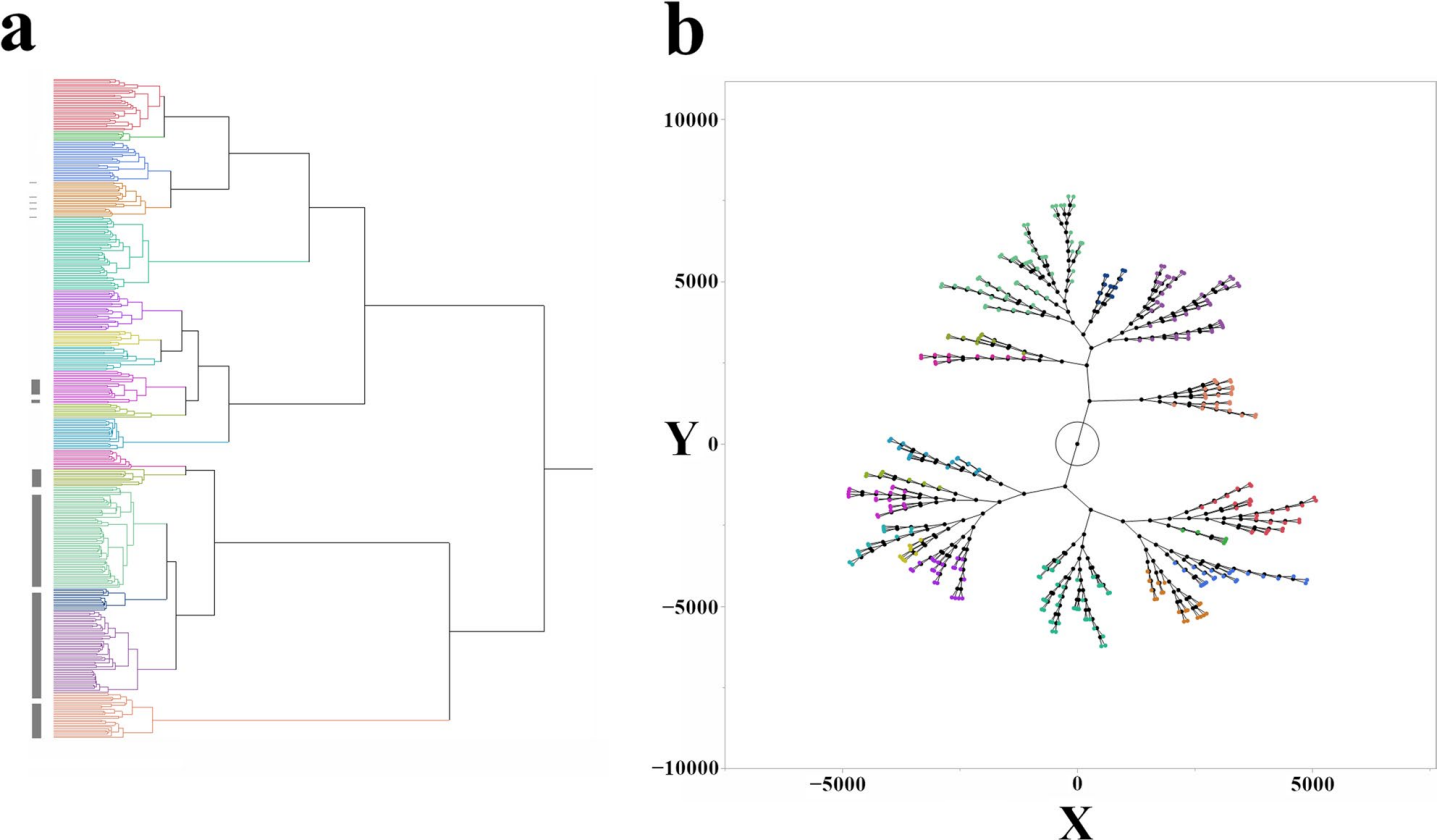
Genetic relationships among sorghum mini core and Senegalese accessions revealed by hierarchical clustering and constellation plot based on SNP markers. **a** Hierarchical cluster dendrogram of 377 sorghum accessions, including the mini core collection and additional Senegalese lines, genotyped at SNP loci. Gray blocks highlight clusters predominantly composed of Senegalese lines. **b** Constellation plot visualizing the relationships among the 17 SNP-based clusters

To investigate the genetic relationships among the mini core and additional Senegal accessions, we performed hierarchical clustering on a combined dataset of 377 accessions (including controls such as SC748-5) using all 297,876 SNP markers (Fig. 3a). Application of the Ward method, which minimizes within-cluster variance, resulted in the algorithmic identification of 17 distinct genetic clusters. Visually, these 17 clusters are nested within six to seven major clades in the dendrogram (Fig. 3a), which illustrates the broader genetic structure of the population. Notably, the Senegalese accessions grouped into approximately six distinct clusters, as indicated by the gray blocks on the dendrogram, highlighting their genetic distinctiveness relative to the mini core collection. Likewise, the relationships between these 17 clusters are also presented in a constellation plot (Fig. 3b) based on the genetic relationships.

### Genome-wide association analysis of geographic origin using Bootstrap Forest models

To further investigate the genetic basis of geographic origin within the sorghum mini-core collection, we performed a GWAS employing a machine learning approach, specifically Bootstrap Forest models. This analysis aimed to identify specific genomic regions (SNPs) most predictive of geographic origin of the accessions, categorized by either country or broader region.

Initial model validation was performed using an 80/20 training/validation split (Table 1). Training set misclassification rates were low (Country: 0.0864; Region: 0.0617), whereas validation misclassification rates were substantially higher (Country: 0.50; Region: 0.3519) (Table 1). Critically, the Generalized R-square for the Country model was negative (-0.534), indicating low model fit on the validation set. While the Region model showed a positive Generalized R-square (0.7866), the low validation accuracy for both geographic origin models suggests that predicting geographic origin, especially at the country level, is challenging with this dataset, likely due to factors such as small sample sizes, the complex history of sorghum dispersal, and potential misclassification of origin in the original germplasm collection. Training set accuracies for the disease resistance models were also high (anthracnose: 0.9753; head smut: 0.9675; downy mildew: 0.9814). However, validation set performance varied considerably across traits. The anthracnose resistance model showed a relatively low but potentially usable validation accuracy of 0.6296 and a low Generalized R-square (0.0548). The validation accuracy for downy mildew resistance was 0.846; however, other metrics such as the Generalized R² (–0.063) and AUC (0.5653) indicate that the model’s predictive performance was limited. This discrepancy is likely due to the imbalanced dataset, in which susceptible accessions (*n* = 163) significantly outnumber resistant ones (*n* = 50), potentially inflating the accuracy score.

**Table 1:** Model performance metrics for Bootstrap Forest models predicting geographic origin and disease resistance

**Trait**	Data set	Sample size (*n*)	Entropy *R*²	Misclassification Rate	AUC	RASE	Generalized *R*²
Country	Training	162	0.7044	0.0864	1	0.60458	0.9922
	Validation	54	-0.073	0.5	0.8234	0.82237	-0.534
Region	Training	162	0.7819	0.0617	0.9999	0.38833	0.978
	Validation	54	0.3605	0.3519	0.8896	0.63063	0.7866
Anthracnose	Training	162	0.6695	0.0247	0.9991	0.22873	0.8061
	Validation	54	0.0303	0.3704	0.6598	0.48742	0.0548
Head smut	Training	162	0.6538	0.0325	0.9966	0.23841	0.7935
	Validation	54	-0.032	0.48	0.5737	0.51023	-0.06
Downy mildew	Training	162	0.6096	0.0186	1	0.23597	0.7372
	Validation	54	-0.042	0.1538	0.5653	0.37079	-0.063

This table presents the results of the initial 80/20 split validation for the Bootstrap Forest models. Metrics are shown for each trait’s training and validation sets, including country of origin, geographic region, anthracnose resistance, head smut resistance, and downy mildew resistance. The metrics reported are: number of samples, entropy R-square, misclassification rate, Area Under the ROC Curve (AUC), Root Average Squared Error (RASE), and generalized R-square

**Fig. 4:**
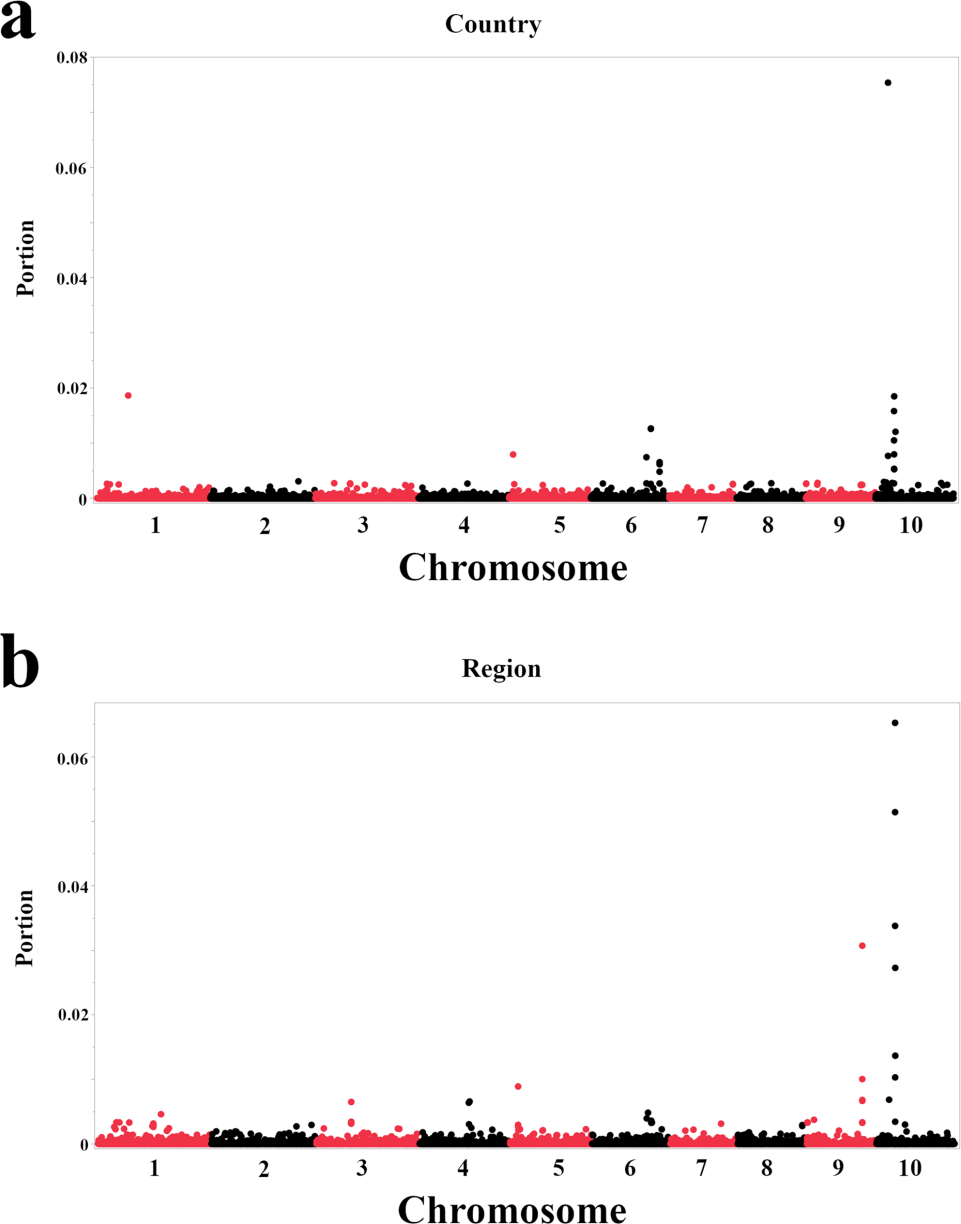
GWAS of geographic origin in sorghum using Bootstrap Forest models. **a** Importance scores (portion) for SNPs associated with the country of origin. **b** Importance scores for SNPs related to broader geographic regions of origin. Importance scores, shown on the y-axis, quantify the contribution of each SNP to the model’s ability to correctly classify accessions based on their geographic origin; a higher score indicates a more significant contribution. The x-axis represents the physical position of each SNP along the ten sorghum chromosomes. The SNPs with the highest importance scores are labeled. Because Bootstrap Forest models do not rely on statistical significance testing, as traditional GWAS methods do, no *p*-value-based significance threshold is shown

**Fig. 5:**
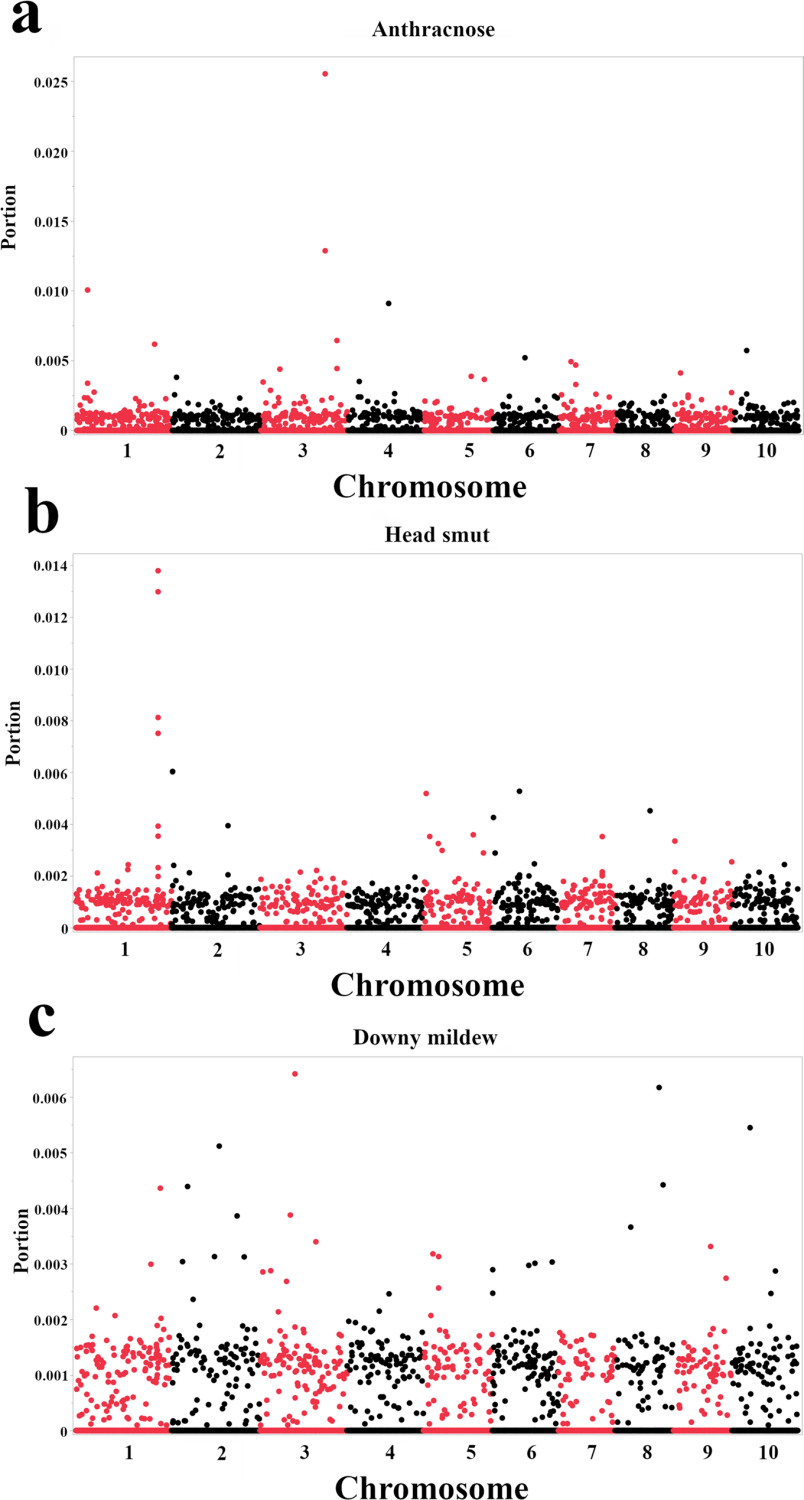
Genome-wide association results for resistance to three sorghum diseases based on Bootstrap Forest models. **a** Anthracnose resistance. **b** Head smut resistance. **c** Downy mildew resistance. Each panel displays a Manhattan plot, where each point represents a single SNP. The x-axis indicates the chromosomal position of the SNP, and the y-axis represents the portion derived from the Bootstrap Forest model. Lead SNPs shown were selected based on the most prominent importance score peaks in the Manhattan plots, as determined by qualitative visual assessment

Given the inconsistent and, in some cases, weak validation performance, we proceeded with extreme caution. While the training set accuracies for all models were high (ranging from 0.9136 to 0.9814), indicating that the models can capture relationships between SNPs and the traits within the training data, the limited validation results suggest that these relationships may not generalize well to new data. Therefore, we focused primarily on the importance scores from Bootstrap Forest models trained on the full dataset (100% of the accessions) to identify potential candidate SNPs and genes. This approach allows us to leverage all available data. However, the results, especially for those traits with low validation performance, should be considered exploratory and require further validation in independent datasets. Based on their importance scores from this final model, the top SNPs associated with geographic origin and disease resistance are discussed below (Fig. 4; Table 2 for geographic origin, Fig. 5; Table 3 for disease resistance).

**Table 2:** Top SNPs associated with geographic origin as identified by Bootstrap Forest models

Origin Model	SNP ID	MAF	Nearest Gene ID	Putative Function	Distance from Gene (bp)	Importance Score
Country	S10_5199488	0.49	*Sobic.010G065500*	bHLH transcription factor PTF1-like	0	0.0753
	S1_16693424	0.42	*Sobic.001G188800*	Cysteine-rich repeat secretory protein	189	0.0186
	S10_7769197	0.45	*Sobic.010G089700*	EREBP-like factor	0	0.0185
	S10_7675418	0.45	*Sobic.010G089300*	DUF6598 domain-containing protein	10,801	0.0158
	S6_53674050	0.4	*Sobic.006G181900*	Aluminium-activated malate transporter	0	0.0126
	S6_53674064	0.4				
	S10_8370408	0.4	*Sobic.010G094100*	No annotation	10,311	0.012
Region	S10_7678887	0.45	*Sobic.010G089300*	DUF6598 domain-containing protein	7,332	0.0652
	S10_7680604	0.45				0.0652
	S10_7675418	0.45			10,801	0.0158
	S9_53912320	0.44	*Sobic.009G186100*	No annotation	49	0.0307
	S9_53912321	0.44				
	S10_7790015	0.44	*Sobic.010G089700*	EREBP-like factor	0	0.0273
	S10_7769197	0.45				
	S10_7715727	0.46	*Sobic.010G089600*	Trehalose 6-phosphate phosphatase	8,601	0.0103
	S5_3709735	0.41	*Sobic.005G040800*	Protein of unknown function (DUF2921)	0	0.0089
	S10_5199488	0.49	*Sobic.010G065500*	bHLH domain-containing protein	0	0.0068
	S9_53923701	0.44	*Sobic.009G186300*	Tyrosine-protein kinase	254	0.0115
	S9_53923509	0.44				
	S4_50278385	0.44	*Sobic.004G158100*	Phosphatidylinositol transfer protein	54	0.0111
	S3_20398659	0.4	*Sobic.003G163301*	No annotation	0	0.0109

The table includes the SNP identifier (SNP ID), Minor Allele Frequency (MAF), the nearest gene and its putative function (if known), the distance in base pairs between the SNP and the nearest gene (0 indicates that the SNP is within the gene), and the importance score (portion) of the SNP in the model. PVE is not calculated by the Bootstrap Forest model. The ‘Importance Score’ shown here quantifies each SNP’s contribution to the model’s predictive accuracy and serves as the analogous metric in this machine learning framework

**Table 3:** Top SNPs associated with resistance to three sorghum diseases as identified by Bootstrap Forest models

Disease	SNP ID	MAF	Nearest Gene ID	Putative Function	Distance from Gene (bp)	Importance Score
Anthracnose	S3_61650227	0.21	*Sobic.003G281450*	Reverse transcriptase zinc-binding domain	1,345	0.0255
	S3_61650258	0.21				
	S1_6061658	0.32	*Sobic.001G078900*	Putative MYB-like DNA-binding protein	0	0.0101
	S4_48780280	0.21	*Sobic.004G154200*	Ankyrin repeat-containing domain	63,053	0.0091
	S3_69017597	0.066	*Sobic.003G375500*	Zinc finger PHD-type domain-containing protein	0	0.0064
	S1_71075125	0.013	*Sobic.001G431800*	Uncharacterized protein DUF292	0	0.0062
	S10_6800171	0.22	*Sobic.010G079900*	H15 domain-containing protein	850	0.0057
Head smut	S1_73522544	0.13	*Sobic.001G459600*	NB-ARC domain-containing protein	222	0.00138
	S1_73523267	0.12			0	0.0013
	S1_73523586	0.21				
	S1_73516778	0.16	*Sobic.001G459500*	Leucine-rich repeats (LRR)-containing protein	270	0.0081
	S2_719163	0.29	*Sobic.002G007700*	Nodulin-like domain-containing protein	0	0.006
	S6_38161717	0.12	*Sobic.006G051700*	NB-ARC domain // WRKY DNA-binding domain	0	0.0053
	S5_1701005	0.19	*Sobic.005G018900*	Phosphoinositide-specific phospholipase C	0	0.0052
	S8_49187870	0.20	*Sobic.008G104801*	DUF4220 domain-containing protein	466	0.0045
Downy mildew	S3_30166225	0.19	*Sobic.003G169966*	3–5 exonuclease	11,050	0.0064
	S8_56098111	0.048	*Sobic.008G133300*	UNC-93 like protein	0	0.0062
	S10_8958624	0.021	*Sobic.010G098932*	Uncharacterized protein	2,458	0.0055
	S2_54739711	0.05	*Sobic.002G173500*	Glycosyltransferase	18,134	0.0051
	S8_58354598	0.14	*Sobic.008G150400*	F-box and WD40 domain protein	0	0.0044
	S2_8823410	0.35	*Sobic.002G082500*	Protein LURP-one-related 15	2,370	0.0044
	S1_74545705	0.011	*Sobic.001G473300*	Protein phosphatase	0	0.0044

A distance of 0 bp indicates the SNP is located within the annotated gene region. PVE is not calculated by the Bootstrap Forest model. The ‘Importance Score’ shown here quantifies each SNP’s contribution to the model’s predictive accuracy and serves as the analogous metric in this machine learning framework

Based on importance scores from the classification models, we identified SNPs most strongly associated with geographic origin (Fig. 4; Table 2). In the country of origin model (Fig. 4a), top-ranking SNPs included S10_5199488 (within *Sobic.010G065500*, a bHLH transcription factor), S1_16693424 (*Sobic.001G188800*, cysteine-rich repeat secretory protein), S10_7769197 (*Sobic.010G089700*, EREBP-like factor), S10_7675418 (*Sobic.010G089300*, DUF6598-domain protein), S6_53674050 and S6_53674064 (both near *Sobic.006G181900*, an aluminium-activated malate transporter), and S10_8370408 (*Sobic.010G094100*, no annotation). In the broader geographic region model (Fig. 4b), a partially overlapping but distinct set of SNPs emerged. Highly ranked markers included S10_7678887, S10_7680604, and S10_7675418 (all within *Sobic.010G089300*, DUF6598-domain protein), S10_7790015 and S10_7769197 (*Sobic.010G089700*, EREBP-like factor), S9_53912320 and S9_53912321 (*Sobic.009G186100*, no annotation), S10_5199488 (*Sobic.010G065500*), and others near genes for trehalose 6-phosphate phosphatase (*Sobic.010G089600*), tyrosine-protein kinase (*Sobic.009G186300*), and phosphatidylinositol transfer protein (*Sobic.004G158100*). The repeated prominence of SNPs on chromosome 10, including S10_5199488, S10_7769197, and S10_7675418, across both models suggests that this chromosome may contain genomic regions contributing to the adaptation and diversification of sorghum across distinct geographic origins.

### Identification of SNPs and candidate genes associated with disease resistance using Bootstrap Forest models

Genotypic and phenotypic data were input into Bootstrap Forest models to identify genomic regions highly associated with resistance to the three major sorghum diseases. For each disease, separate models were trained using the same SNP markers, resulting in the identification of SNPs with outstanding importance scores for each disease, which suggests their significant contribution to predicting disease resistance. The importance scores and details of the top SNPs for each disease, including their nearest genes and associated putative functions, are presented in Fig. 5; Table 3.

For anthracnose resistance, the SNPs with the highest importance scores were S3_61650227 and S3_61650258 (nearest gene: *Sobic.003G281450*, Reverse transcriptase zinc-binding domain-containing protein), S1_6061658 (nearest gene: *Sobic.001G078900*, Putative Myb-like DNA-binding protein), S4_48780280 (nearest gene: *Sobic.004G154200*, Ankyrin repeat-containing domain), S3_69017597 (nearest gene: *Sobic.003G375500*, Zinc finger PHD-type domain-containing protein), S1_71075125 (nearest gene: *Sobic.001G431800*, Uncharacterized protein DUF292), and S10_6800171 (nearest gene: *Sobic.010G079900*, H15 domain-containing protein) (Fig. 5a).

For head smut resistance, the top SNPs were S1_73522544, S1_73523267, and S1_73523586 (nearest gene: *Sobic.001G459600*, NB-ARC domain-containing protein), S1_73516778 (nearest gene: *Sobic.001G459500*, Leucine-rich repeats-containing protein), S2_719163 (nearest gene: *Sobic.002G007700*, Nodulin-like domain-containing protein), S6_38161717 (nearest gene: *Sobic.006G051700*, NB-ARC domain // WRKY DNA -binding domain), S5_1701005 (nearest gene: *Sobic.005G018900*, Phosphoinositide-specific phospholipase C), and S8_49187870 (nearest gene: *Sobic.008G104801*, DUF4220 domain-containing protein) (Fig. 5b).

For downy mildew resistance, the top SNPs were S3_30166225 (nearest gene: *Sobic.003G169966*, 3–5 exonuclease), S8_56098111 (nearest gene: *Sobic.008G133300*, UNC-93 like protein), S10_8958624 (nearest gene: *Sobic.010G098932*, Uncharacterized protein), S2_54739711 (nearest gene: *Sobic.002G173500*, Glycosyltransferase), S8_58354598 (nearest gene: *Sobic.008G150400*, F-box and WD40 domain protein), S2_8823410 (nearest gene: *Sobic.002G082500*, Protein LURP-one-related 15), and S1_74545705 (nearest gene: *Sobic.001G473300*, Protein phosphatase) (Fig. 5c). The identification of multiple SNPs with high importance scores for each disease supports the hypothesis that resistance to anthracnose, head smut, and downy mildew in sorghum is likely controlled by multiple genes or genomic regions, exhibiting a polygenic inheritance pattern.

## Discussion

This study examines the intricate relationship between geographic origin, genetic diversity, and disease resistance in sorghum. We primarily analyzed the global mini core collection, which represents a broad spectrum of sorghum diversity and additional Senegalese lines [30]. This collection has been valuable for assessing resistance to various diseases, specifically anthracnose, leaf blight, and rust [31]. To enhance our assessment of genetic diversity, we included SNP data from an additional set of Senegalese accessions, which allowed for a more comprehensive evaluation of the relationship between geographic origin, genetic variation, and disease resistance in sorghum. Leveraging a combination of phenotypic evaluations for resistance to three major diseases, extensive genotypic data from 297,876 SNP markers, and an advanced machine learning approach, we aimed to better understand the genetic architecture of sorghum disease resistance and to identify genomic regions associated with geographic adaptation.

### Phenotypic variation and genetic diversity

Our analyses revealed complex relationships among geographic origin, genetic diversity, and disease resistance profiles in sorghum. While the *t*-SNE analysis (Fig. 2a) of disease resistance phenotypes in mini core lines revealed some clustering, this clustering did not directly correlate with geographic origin, as each cluster contained accessions from various countries. This suggests that, although adaptation to local environments, including pathogencapable of identifying complex associations between genotype and phenotype pressures [42], likely plays a role, the observed patterns of disease resistance are not solely explained by geographic proximity. The loading plot from the PCA (Fig. 2b) further clarified the contribution of each disease to the overall phenotypic variation. The loading plot showed that, for each disease, resistance and susceptibility loaded in opposite directions (as expected for complementary binary variables). While all three diseases contributed significantly to the observed variation, downy mildew resistance had the strongest influence on PC2, whereas anthracnose resistance had the most significant influence on PC1. Moreover, this study also found statistical significance in genetic distances based on the originating country or geographic region (ANOVA, *p* < 0.0001) (Fig. S1), indicating that genetic structure differs significantly across countries and regions [17]. Furthermore, hierarchical clustering based on SNP data (Fig. 3a and b) revealed that the Senegalese accessions formed approximately six distinct clusters, largely separate from the mini core collection. This clear genetic separation between the Senegalese lines and most of the mini core accessions points to substantial genetic distinctiveness of the Senegalese panel relative to the mini core collection. This pattern is consistent with historical and environmental factors shaping local diversity, but the drivers cannot be resolved here.

### GWAS of geographic origin and disease resistance

To gain deeper insights into the genetic foundations of the observed geographic patterns, we conducted a GWAS employing Bootstrap Forest models, a powerful machine-learning approach capable of identifying complex associations between genotype and phenotype [43]. This analysis aimed to identify SNPs most predictive of geographic origin of the accessions, using both country (56 countries) and broader regional (13 regions) classifications. While the training set accuracies for the models were high, the validation performance varied considerably between the two geographic models. Notably, the model for predicting the country of origin showed poor validation performance, with a negative Generalized R-square (-0.534), indicating a low model fit. In contrast, the model for the broader geographic regions performed better, achieving a positive Generalized R-square (0.7866), although its validation accuracy was still considerably lower than its training accuracy. This discrepancy between training and validation set accuracy, particularly for the country-level model, suggests that predicting specific geographic origins from SNP data in this dataset is challenging and potentially requires a larger dataset. As regional predictions were comparably more accurate, this could be due to the difference in the number of classification categories (13 regions vs. 56 countries). While these models exhibit good explanatory power within the training dataset, their limited validation performance, including negative generalized R² values for the head smut and downy mildew models and unstable validation metrics for downy mildew (e.g., generalized R² < 0 and an AUC near 0.5), indicates that these findings should be interpreted as exploratory and hypothesis-generating rather than predictive. Consequently, we focused our interpretation on the SNP importance scores derived from Bootstrap Forest models trained on the full dataset. This approach allows us to leverage all available information to identify potential candidate genes. The analysis identified numerous SNPs with high importance scores, indicating their substantial contribution to distinguishing between accessions from different geographic locations (Fig. 4; Table 2). When using the country of origin as the target variable, the most significant SNP was S10_5199488, located within a gene (*Sobic.010G065500*) encoding a bHLH transcription factor (Table 2) [44]. This family of transcription factors plays diverse roles in plant development and responses to environmental stimuli and may be a potential link between this genomic region and adaptation to specific ecological conditions within different countries [44–46]. Other important SNPs for the country of origin were found on chromosomes 1, 6, and 10 (Table 2), further accentuating the polygenic nature of geographic adaptation. For instance, the SNP S1_16693424 is located near the gene *Sobic.001G188800*, which encodes a cysteine-rich repeat secretory protein (CRRSP). CRRSPs are a family of proteins known to be involved in various processes, including plant development [47], stress responses [47, 48], and signaling. In *Arabidopsis*, CRRSPs are induced by pathogen infection and treatment with reactive oxygen species or salicylic acid [49]. Similarly, when the analysis was performed using the broader geographic region as the target variable, a distinct set of SNPs emerged as highly important, with several located on chromosome 10 (Fig. 4b; Table 2). Several of the most important SNPs for the region of origin, including S10_7675418, are located near a gene (*Sobic.010G089300*) encoding a DUF6598 domain-containing protein. The precise function of the gene in sorghum remains to be explored, but a DUF6598 domain has been identified as a candidate gene for powdery mildew resistance in wheat [50]. Additionally, SNP S10_7790015, also found to be important for predicting the region of origin, is located near a gene encoding an EREBP-like factor, further highlighting the potential role of transcription factors in sorghum’s adaptation to abiotic and biotic stresses, such as drought [51] and fungal pathogens [52], which can vary significantly across broad geographic regions. The prominence of Chromosome 10 in geographic adaptation, particularly the identification of a DUF6598 domain previously linked to fungal resistance, motivates a testable hypothesis: selective pressures contributing to geographic differentiation in this region may include adaptation to location-specific pathogen communities. Accordingly, some loci associated with geographic origin could reflect signatures of spatially structured biotic selection, although this interpretation is hypothesis-generating and requires independent validation. This pattern was also observed in *Medicago*, where SNP-based clustering and GWA analysis using machine learning highlighted the importance of chromosome 8 in distinguishing geographic origins [53]. Together, these results illustrate the utility of machine learning approaches, such as Bootstrap Forest, for prioritizing genomic regions associated with plant origin and local adaptation [53].

Building upon the insights into geographic adaptation, we extended our investigation to explore the genetic architecture of resistance to three major sorghum diseases: anthracnose, head smut, and downy mildew. Employing the same Bootstrap Forest modeling approach, we again trained separate models for each disease using identical SNP markers. Numerous SNPs possessed high importance scores in each model, displaying their substantial contribution to predicting disease resistance phenotypes (Fig. 5; Table 3). The most important SNPs (selected as the lead markers from the most prominent signal peaks in our analysis) for anthracnose resistance were found on chromosomes 1, 3, 4, and 10, while those for head smut resistance were located on chromosomes 1, 2, 5, 6, and 8. Similarly, SNPs associated with downy mildew resistance were identified on chromosomes 1, 2, 3, 8, and 10. This wide distribution of important SNPs across multiple chromosomes strongly supports the hypothesis that resistance to these diseases is a complex, polygenic trait. The identification of candidate genes from diverse biological pathways suggests that this resistance is driven by multiple molecular mechanisms.

For anthracnose resistance, two of the top SNPs, S3_61650227 and S3_61650258, are located near a gene (*Sobic.003G281450*) encoding a reverse transcriptase zinc-binding domain-containing protein. While the precise role of this gene in anthracnose resistance is unknown, its zinc-binding domain is of particular interest. Zinc-binding proteins have been implicated in plant defense responses; for instance, a zinc-binding citrus protein metallothionein can act as a plant defense factor [54]. In *Arabidopsis*, zinc has been shown to trigger signaling mechanisms and defense responses that promote resistance to *Alternaria brassicicola* [55]. Moreover, a zinc metalloprotease in *Fusarium graminearum* targets a wheat zinc-binding protein, contributing to the pathogen’s overall virulence [56]. These findings suggest that zinc-binding proteins, such as the one encoded by *Sobic.003G281450*, may play a role in sorghum’s defense against anthracnose. Interestingly, a linear mixed model identified this region as a top candidate SNP for anthracnose resistance in our previous traditional GWAS analysis [6]. This further augments the argument that this region, and potentially the zinc-binding domain-containing protein encoded by *Sobic.003G281450* (or nearby genes), is involved in sorghum’s defense response. Other genes involved in diverse functions were found near the top SNPs, including those encoding a putative MYB-like DNA-binding protein, an ankyrin repeat-containing domain, and a zinc finger PHD-type domain-containing protein.

For head smut resistance, the most important SNP, S1_73522544, is located near the gene *Sobic.001G459600*, which encodes a NB-ARC domain-containing protein, a key component involved in plant disease resistance [57]. NB-ARC domains are the central engine of the largest class of plant resistance (R) proteins, which typically work in concert with LRR domains that are involved in pathogen recognition. Curiously, another LRR-containing protein encoded by the gene *Sobic.001G459500* was also identified as a top candidate for head smut resistance in our previous traditional GWAS analysis [6]. This gene is located near the SNP S1_73516778, further supporting the importance of LRR-containing proteins in sorghum’s defense against head smut. In addition to this LRR protein and other NB-ARC domain proteins, other genes associated with head smut resistance identified in this study suggest a complex interplay between defense signaling and recognition mechanisms. These include genes encoding an NB-ARC domain-containing protein and a protein with both NB-ARC and WRKY DNA-binding domains. NB-ARC domains are characteristic of intracellular immune receptors (R proteins) that recognize pathogen effectors and trigger defense responses, while WRKY transcription factors are key regulators of defense gene expression [58]. Notably, a recent study in chickpeas demonstrated a direct physical interaction between a CC-NB-ARC-LRR protein and a WRKY transcription factor, promoting resistance to Fusarium wilt [59].

Finally, for downy mildew resistance, one of the top SNPs, S2_54739711, is located near *Sobic.002G173500*, a gene encoding a glycosyltransferase protein. Glycosyltransferases are involved in the biosynthesis of various cell wall components, and modifications to the cell wall can influence pathogen penetration and spread [60]. We also identified an F-box protein as a potential candidate, which is noteworthy as F-box proteins are known to play roles in plant defense, and multiple studies similarly found an F-box protein gene associated with fungal resistance in sorghum [61–63]. The identification of an F-box protein as a candidate for downy mildew resistance, coupled with previous reports implicating F-box proteins in anthracnose resistance, suggests a potential convergent defense strategy in sorghum. We hypothesize that the ubiquitin-proteasome pathway, mediated by F-box proteins, may act as a versatile defense hub, enabling the plant to recognize and target key pathogenic proteins from multiple, distinct fungal lineages.

The diversity of these candidate genes suggests that resistance to each disease is not only polygenic but also involves a complex interplay of various defense mechanisms, potentially entailing pathogen recognition [64], signal transduction [65], cell wall modification [66], and other metabolic processes. Additional research incorporating aspects such as gene expression studies and functional characterization is needed to elucidate the precise roles of these candidate genes in conferring resistance to anthracnose, head smut, and downy mildew in sorghum.

### Implications for sorghum breeding and future research

It is important to acknowledge a limitation regarding the generalizability of the disease resistance findings. The phenotypic evaluations used in this study were based on pathogen isolates relevant to a specific geographic region (Southern U.S.). While this is valuable for understanding resistance, the prevalence, virulence, and genetic diversity of *C. sublineola* (anthracnose), *S. reilianum* (head smut), and *P. sorghi* (downy mildew) can vary across different geographic regions. Still, the identified loci and genes will remain valuable candidates for further investigation. Second, our germplasm panel did not include accessions from key centers of sorghum origin, such as Ethiopia and Sudan, which should be a focus for future diversity studies. The pronounced genetic distinctiveness of the Senegalese accessions suggests they are not merely an additional source of diversity, but a reservoir of locally adapted traits. We hypothesize that these accessions harbor novel resistance alleles, particularly against West African pathotypes of anthracnose and head smut, which may not be present in the broader mini-core collection. Consequently, phenotyping and conducting a targeted GWAS on this sub-population is a critical next step to introduce unique and potentially durable resistance into breeding programs. Another key methodological consideration is our conversion of quantitative disease scores into a binary qualitative trait (Resistant/Susceptible). We acknowledge the valid concern that this results in a loss of statistical information. However, the theoretical distinction between quantitative and qualitative resistance is not always strict, and analyzing disease traits as binary outcomes is a valid approach in genetic studies [67, 68]. This study chose this approach for two primary reasons. First, the binary classification corresponds to a clear biological threshold that is highly relevant for breeding purposes. Second, the Bootstrap Forest model we employed is a powerful classification algorithm that excels at identifying major loci, including higher-order and non-linear effects, associated with such distinct binary outcomes [69, 70]. While we believe our approach is valid for the goals of this study, we recognize that a future analysis using the full quantitative data could potentially reveal additional genomic regions with more subtle effects on the resistance response.

The candidate loci provide targets for marker development and marker-assisted selection (MAS) [71], and they help prioritize genes for future functional validation, including gene-editing approaches such as CRISPR-Cas9 [72]. By focusing on these genes, previously known candidates, and their associated pathways, breeders can more efficiently select and pyramid resistance genes, developing varieties with durable and broad-spectrum resistance. For instance, identifying LRR-containing proteins as potential players in head smut resistance provides a clear avenue for targeted breeding, as these proteins are known to be involved in pathogen recognition. Similarly, the association of glycosyltransferases with downy mildew resistance suggests that modifying cell wall composition could be a promising strategy for enhancing resistance to this disease. However, follow-up studies need to validate the functional roles of these candidate genes through further research.

To facilitate these breeding applications, we have provided the physical location and MAF for each top SNP in Tables 2 and 3. Within our machine learning framework, the ‘importance score’ for each marker serves a role analogous to the PVE in traditional linear models, quantifying its contribution to predictive accuracy and helping to prioritize these targets. Furthermore, future studies should continue to explore the genetic diversity of underrepresented germplasm collections, as they likely contain a wealth of untapped genetic variation for disease resistance and other valuable traits.

To directly facilitate breeding applications, our results allow for the strategic selection of parental lines that are both resistant and genetically diverse. By cross-referencing the phenotypic data with the cluster analysis (Supplementary Table 1), breeders can identify valuable candidates for further evaluation. This strategy of crossing a parent that is resistant to one disease from a specific genetic cluster with another parent resistant to a different disease from a distinct cluster is a powerful approach for pyramiding multiple resistance genes and enhancing durable resistance in new sorghum varieties.

## Conclusion

This study employed a comprehensive approach, integrating phenotypic evaluations, high-density SNP genotyping, and machine learning analyses to investigate the genetic architecture of disease resistance and geographic origin in sorghum. Our findings confirm that the geographic origin has a profound influence on sorghum’s genetic diversity and disease resistance profiles. The inclusion of a genetically distinct Senegalese collection substantially broadened this diversity, emphasizing the importance of exploring underrepresented germplasm to identify valuable traits.

We identified numerous SNPs associated with geographic origin and disease resistance that were distributed across multiple chromosomes, highlighting the polygenic nature of these traits. For geographic adaptation in particular, chromosome 10 appears to be a potential hotspot for geographic adaptation based on SNP associations identified in this study. We identified several candidate genes located near the most important SNPs, consisting of those encoding transcription factors (bHLH and EREBP-like factors), a cysteine-rich repeat secretory protein, and a DUF6598 domain-containing protein. These findings suggest that diverse molecular mechanisms, including transcriptional regulation and stress responses, as well as potentially novel pathways, contribute to sorghum’s adaptation to varying environments.

For disease resistance, our analysis revealed a complex, polygenic architecture with important SNPs distributed across multiple chromosomes. We identified candidate genes associated with resistance to anthracnose, head smut, and downy mildew, including those encoding zinc-binding proteins, LRR-containing proteins, F-box proteins, glycosyltransferases, and others involved in various cellular processes. Interestingly, several candidate genes identified in this study were also identified in our previous traditional GWAS analysis, further strengthening the evidence for their involvement in disease resistance.

Our identification of candidate genes near top-ranked SNPs associated with disease resistance and geographic origin provides a foundation for hypothesis-driven functional studies and precision breeding. Notably, genes encoding zinc-binding proteins, LRR- and NB-ARC-domain proteins, F-box components, and stress-related transcription factors emerged as notable candidates across multiple disease models and geographic contrasts, providing a preliminary foundation for future functional studies. While the machine learning-based GWAS models employed here are exploratory and require further validation, the convergence of several key loci with known defense pathways underscores their potential biological relevance. These findings suggest potential targets for marker-assisted selection and genome editing, pending further validation. More broadly, this study highlights the utility of integrating machine learning with high-density genotyping in dissecting complex, polygenic traits—an approach that may be extended to other crops facing similar challenges under climate and pathogen stress.

## Supplementary Information

Supplementary Material 1. Fig. S1 Genetic distance among sorghum mini core accessions based on SNP data. The bar chart shows the average genetic distance among sorghum mini core accessions, calculated from 297,876 SNP markers using the IBS method. Distances are grouped by resistance or susceptibility to three diseases (anthracnose, head smut, and downy mildew), by country of origin, and by broader geographic region. Error bars indicate the standard error of the mean.

Supplementary Material 2.

## Data Availability

The dataset supporting the conclusions of this article is included within the article and its additional files. The list of all 377 accessions, their origins, phenotypic data, and assigned genetic clusters is provided in Supplementary Data 1. The genotypic variant (SNP) data generated and analyzed during the current study have been deposited in the European Variation Archive (EVA) at EMBL-EBI under project accession number PRJEB108543 (https://www.ebi.ac.uk/eva/?eva-study=PRJEB108543).
