# Supplementary figures and images for: A machine learning approach to genome-wide association mapping of disease resistance and geographic origin in sorghum
Source: BMC Plant Biol. 2026 Feb 28;26:614. doi: 10.1186/s12870-026-08468-z (PMC13059599; doi:10.1186/s12870-026-08468-z)

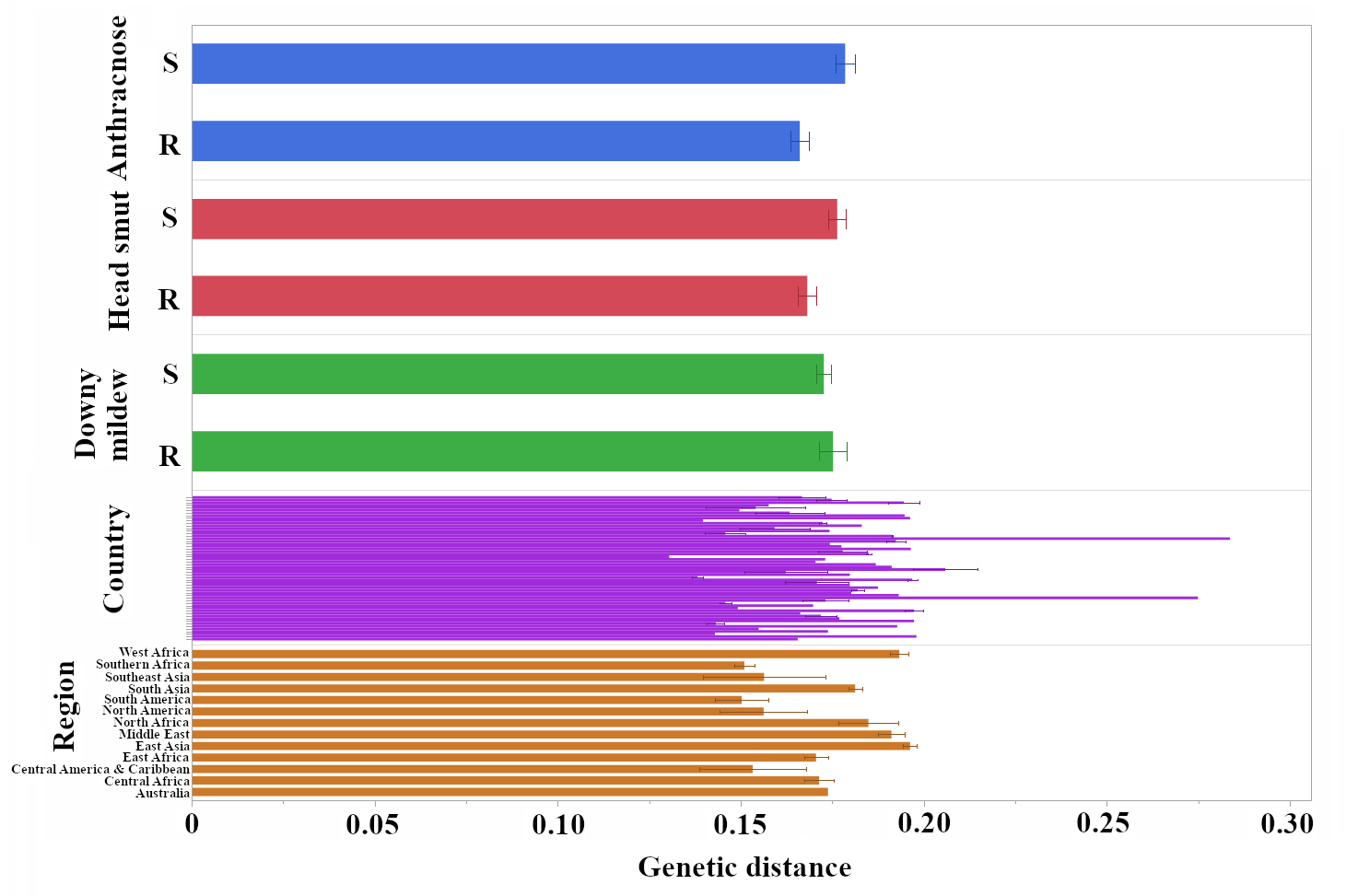

Supplement: Supplementary file 1 — Supplementary Material 1. Fig. S1 Genetic distance among sorghum mini core accessions based on SNP data. The bar chart shows the average genetic distance among sorghum mini core accessions, calculated from 297,876 SNP markers using the IBS method. Distances are grouped by resistance or susceptibility to three diseases (anthracnose, head smut, and downy mildew), by country of origin, and by broader geographic region. Error bars indicate the standard error of the mean. [file 12870_2026_8468_MOESM1_ESM.png]
